# How Ethical Leadership Promotes Knowledge Sharing: A Social Identity Approach

**DOI:** 10.3389/fpsyg.2021.727903

**Published:** 2021-10-14

**Authors:** Wei-Li Wu

**Affiliations:** Department of International Business, Chien Hsin University of Science and Technology, Taoyuan, Taiwan

**Keywords:** ethical leadership, knowledge sharing, group identification, relational identification, organizational identification, social identity theory

## Abstract

The aim of this study is to investigate the associations among ethical leadership, group identification, relational identification, organizational identification, and knowledge sharing. This study conducted a survey in Taiwan to collect the data. The administrative group members of schools were invited to participate in this study. The sample included 510 participants, and the hypotheses were tested by using the path analysis and bootstrapping methods in the Mplus program to examine how ethical leadership influences knowledge sharing, through various means of identification. The results of this study show that ethical leadership has both a direct and indirect effect on knowledge sharing. There are two mediating paths in the ethical leadership-knowledge sharing relationship. Firstly, group identification mediates the relationship between ethical leadership and knowledge sharing. Secondly, ethical leadership has an influence on knowledge sharing by means of increased relational and organizational identification. This is a pioneering article that explores the psychological mechanism between ethical leadership and knowledge sharing, using the social identity approach. This study has shown that the social identity theory (SIT) is a useful and promising perspective for future research studies on ethical leadership-knowledge sharing.

## Introduction

In the knowledge economy, knowledge is one of the most important assets and a critical source of competitive advantage. Most companies are eagerly accumulating a stock of knowledge by using well-established knowledge management. However, when there is no knowledge sharing among employees, it is difficult to achieve knowledge management (Wu and Lee, 2017). As a result, knowledge sharing is considered to be an important issue in knowledge management research.

In the past two decades, researchers have put a lot of effort into exploring the antecedents of knowledge sharing (Wang and Noe, 2010). Currently, the extant research has revealed several environmental factors that can effectively promote the knowledge sharing of employees, such as the reward/incentives system, culture, leadership, team characteristics, etc. (Cabrera et al., 2006; Hsu et al., 2011; Liu and DeFrank, 2013). Of these antecedents, the influence of leadership has increasingly received the attention of researchers in recent years. Studies have begun to discuss how the leadership style of the immediate supervisors of employees impacts their knowledge-sharing performance. This is not surprising, because immediate leaders can always have a significant impact on the behavior of their subordinates. Regarding the influence of immediate leadership on knowledge sharing, most extant studies argue that positive leadership, like empowering and transformational leadership, has a positive impact on knowledge sharing (Liu and DeFrank, 2013; Wu and Lee, 2017), and negative leadership, such as abusive supervision, has a negative effect on knowledge sharing (Wu and Lee, 2016; Lee et al., 2018).

Although knowledge sharing can also basically be considered as a moral challenge, leadership-knowledge sharing research is rarely conducted under a moral lens (Bavik et al., 2018). At the workplace, if employees tend to hide their knowledge instead of sharing, it leads to negative influences on employees’ and organizations’ productivity and performances (Abdullah et al., 2019; Anser et al., 2021). Moreover, it also jeopardizes employees’ and organizations’ learning and development (Usman et al., 2019). Previous studies have claimed that knowledge sharing is an important moral issue (Lin, 2007; Lin and Joe, 2012). If there is a lack of willingness to engage in knowledge sharing by most employees, companies might lose their competitive advantage. Therefore, successful knowledge sharing is vital for a company’s survival and sustainable operations. Bavik et al. (2018) first point out that it is necessary and important to employ a moral lens, in order to explore how to foster knowledge sharing, and that ethical leadership is an essential antecedent of knowledge sharing. Although numerous previous studies have addressed how leadership styles influence knowledge sharing, only a few focus on the impact of ethical leadership. So far, the positive impact of ethical leadership on knowledge sharing has been supported (Lei et al., 2019; Bhatti et al., 2020). However, the underlying mechanism between ethical leadership and knowledge sharing has not been well-understood. According to previous studies, employees’ identifications could be important mediators between leadership and knowledge sharing (Carmeli et al., 2011; Liu and Li, 2018). Meanwhile, previous studies have argued that ethical leadership could sharpen employees’ identification (Walumbwa et al., 2011; Zhu et al., 2015). In other words, employees’ identifications could be important mediators between ethical leadership and knowledge sharing. However, there are few studies to explore what role employees’ identifications play in the relationship between leadership and knowledge sharing. In order to fill this research gap, this study draws on the social identity theory (SIT) (Tajfel, 1982; Ashforth and Mael, 1989) to investigate the identity-mediation mechanism that links ethical leadership and knowledge sharing. In particular, this study will explore the knowledge-sharing behavior of employees within the context of work groups, because they are the most common team units in a company and also the place where knowledge sharing occurs most often (Wu and Lee, 2017).

It is reasonable to apply the perspective of SIT to the relationship between ethical leadership and knowledge sharing. Firstly, in ethical leadership literature, researchers argue that SIT is an emerging and promising theoretical perspective from which to explore the underlying mechanism linking ethical leadership and the attitudes and behavior of the followers (Brown and Mitchell, 2010; Walumbwa et al., 2011; Zhu et al., 2015). Since ethical leadership normally displays positive and prestigious images, and employees usually want to be associated with such identities, leaders with a high level of ethical leadership can play an important role in developing the followers’ identification and then influencing their attitudes and behaviors (Brown and Mitchell, 2010). Secondly, according to the social identity model of leadership, scholars argue that leaders have a huge influence on building the identification of employees, and then influencing their attitudes and behavior (Hogg, 2001; van Knippenberg and Hogg, 2003; Epitropaki et al., 2017). Therefore, shaping the identification of employee is an important psychological mechanism that can be used to connect leadership (e.g., ethical leadership) and the desired organizational behavior (e.g., knowledge sharing). The main purpose of this study is to explore the underlying mechanism between ethical leadership and knowledge sharing, within a group context. Drawing on SIT, this study uses group identification, relational identification, and organizational identification as the mediators. According to SIT, since group members’ group identification and relational identification would be easily developed and presented in the context of work group (Sluss and Ashforth, 2008; Zhang et al., 2014), these two identification are firstly chosen as the mediators. Furthermore, because previous studies have argued that relational identification is positively related to organizational identification (Carmeli et al., 2011; Sluss et al., 2012), this study also includes organizational identification as the mediator.

**FIGURE 1:**
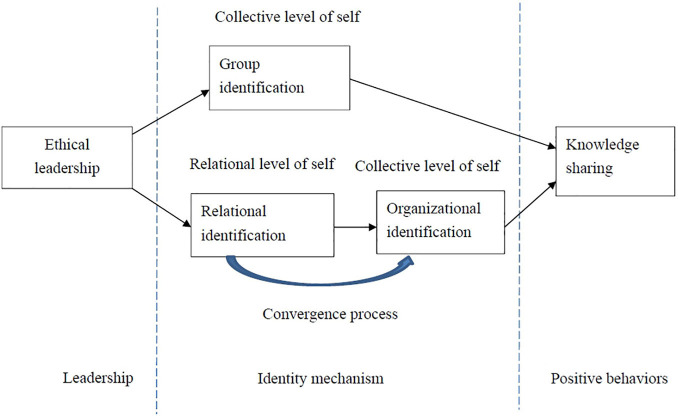
Research framework.

According to SIT, this study argues that ethical leadership will influence knowledge sharing by means of two types of social identity paths. Firstly, this study expects that leadership has a positive impact on knowledge sharing through group identification, as members with high levels of group identification will take the group’s interests into account (van Knippenberg et al., 2004) and then engage in knowledge sharing. Secondly, the research on SIT has shown that relational identification is positively related to organizational identification (Sluss et al., 2012); based on SIT, organizational identification is also supposed to be positively connected to knowledge sharing, because members with high levels of organizational identification tend to share knowledge, in order to benefit their organizations. Thus, this study argues further that there is a serial mediation effect of ethical leadership on knowledge sharing *via* relational and organizational identification. The research framework of this study is presented as Figure 1.

This study will provide some important theoretical contributions to the relevant literature. Firstly, with regard to the research on the antecedents of knowledge sharing, although many studies have explored the relationship between leadership and knowledge sharing, few have revealed how leadership influences knowledge sharing under a moral lens. By exploring the underlying identity mechanism that links ethical leadership and knowledge sharing, this study extends the limited extant knowledge-sharing research on how moral leadership (i.e., ethical leadership) is linked to knowledge sharing. Especially, we could have a more complete understanding of the identity-mediation mechanism between ethical leadership and knowledge sharing. Secondly, previous research on ethical leadership has been applied mainly to the theoretical perspectives of the social learning and social exchange theories to explain how ethical leaders influence the psychological mechanisms of their employees and, in turn, to achieve positive organizational behaviors. By applying SIT, this study will enrich the theoretical development of ethical leadership research. Thirdly, individuals usually identify with multiple social referents in the workplace. By investigating the dual-identity mechanism of identification, and the convergence process of identification within the relationship between ethical leadership and knowledge sharing, this study extends the usefulness of SIT in a new and important research stream (i.e., knowledge-sharing research).

## Literature Review

### Social Identity Theory and Knowledge Sharing

Organizational researchers have shown much interest in the concepts of identity and identification. For an employee, identity refers to what something is; and identification is the extent to which the employee includes that identity as a partial identification of self. SIT is a major theoretical perspective for discussing how individuals connect themselves to, and identify with, various referents in an organization; the referents could be the organization, the group and the relationships that form the organizational, group, and relational identification, respectively (Sluss and Ashforth, 2008). Basically, individuals can have multiple referents at the same time, so individuals will have simultaneous multi-identifications (Sluss and Ashforth, 2008; Epitropaki et al., 2017). Furthermore, different types of identification can cooperate and converge (Sluss and Ashforth, 2008; Carmeli et al., 2011; Sluss et al., 2012). According to SIT, when individuals define “self” in terms of their collective level, they also take the interests of the collective to heart (van Knippenberg et al., 2004). When applying this concept to this study, if an employee can include the group or organization in his or her self-concept (i.e., the collective level of self), such as group and organizational identification, the employee will be willing to engage in knowledge sharing, because he or she already perceives a sense of unity with, or belonging to, the group or organization.

### Knowledge Sharing

In general, managers want their employees to share their knowledge, as it will definitely benefit their companies. But, employees would not engage in knowledge sharing without any hesitation or concern. If an employee always shares his/her tacit knowledge with others, but the others are opportunistic in that they are only learning, without sharing, and acting as free-riders, then the knowledge sharer faces not only the cost of his/her time spent teaching other people, but it also decreases his/her chances for advancement, or even increases the possibility of losing his/her job. Thus, previous studies have put a lot of effort into exploring the antecedents of knowledge sharing, and leadership was found to be an important determinant of knowledge sharing (Srivastava et al., 2006; Liu and DeFrank, 2013; Lee et al., 2018).

Regarding the leadership-knowledge sharing literature, positive leadership, such as empowering leadership and transformational leadership, has been proved to have a positive influence on knowledge sharing. For example, empowering leadership is positively related to knowledge sharing, at both the group and cross levels (Srivastava et al., 2006; Wu and Lee, 2017). Transformational leadership also has a positive cross-level influence on knowledge sharing (Liu and DeFrank, 2013). It is obvious that leadership is a key determinant for knowledge sharing. However, Bavik et al. (2018) point out that knowledge sharing is also a moral challenge, because if most employees within an organization do not willingly engage in knowledge sharing, it will result in poor competition for the organization and a possible shutdown (Lin, 2007; Bavik et al., 2018). Thus, researchers have argued that it is necessary to discuss the impact of leadership on knowledge sharing under a moral lens, and that ethical leadership is the appropriate leadership style with which to present moral leadership (Bavik et al., 2018; Lei et al., 2019). Drawing on SIT, this study explores the identity mechanism that underlies the relationship between ethical leadership and knowledge sharing.

### Ethical Leadership

With more and more corporate scandals occurring, scholars have shown an increasing concern for the moral side of a leader. As a result, ethical leadership is presented and attracts much of the researchers’ attention (Brown et al., 2005; Brown and Mitchell, 2010; Ko et al., 2018). Ethical leadership is defined as the demonstration of normative behavior through personal action and interpersonal relationship, and promote this behavior to followers through two-way communication, reinforcement, and decision-making (Brown et al., 2005). In essence, ethical leadership could be described well by using two dimensions, namely, the moral person and the moral manager (Treviño et al., 2000, 2003; Brown and Mitchell, 2010). The moral person dimension refers to the qualities of the ethical leader as a person. Strong moral persons are considered to be honest, principled, trustworthy, and approachable; they show a concern for their followers and treat them fairly. The moral manager dimension describes how ethical leaders use their power to create a moral environment in the workplace. Ethical leaders are moral role models in organizations; they set and communicate clear ethical standards to their followers. Furthermore, they implement both rewards and punishments, in order to ensure that followers really take the ethical standards to heart.

According to the moral characteristics of ethical leadership, this study predicts that ethical leaders will have a positive influence on the employees’ knowledge sharing within a group. Since the ethical leader of a group is honest, principled and trustworthy, followers will tend to trust him or her in the work environment. Previous studies have shown that ethical leadership can foster the followers’ perception of trust (Newman et al., 2014) and psychological safety (Walumbwa and Schaubroeck, 2009), while it can also decrease the employees’ fear of retaliation (Mayer et al., 2013). In this situation, members will be more likely to share their knowledge with their co-workers because they are not be worried about losing their job once they have shared their unique and valuable knowledge with others. Ethical leaders should also implement both rewards and punishments, in order to ensure ethical standards are set in the workplace (Brown et al., 2005; Brown and Mitchell, 2010). Supposedly, ethical leaders should honestly reward knowledge sharers and punish knowledge hoarders. Thus, employees tend to be less afraid of free riders who only receive knowledge, without contributing. In summary, the ethical leader of a group can establish a friendly and fair group environment to solve the social dilemma of knowledge sharing, and can thus encourage members to share their knowledge. Therefore, this study predicts the following:

Hypothesis 1: Ethical leadership is positively related to the employees’ knowledge sharing.

### Ethical Leadership and Group Identification

As depicted in Figure 1, according to SIT, this study further proposes that the effect of ethical leadership on the knowledge sharing of employees is mediated by group identification. Group identification is one kind of social identification (Zhang et al., 2014), which refers to the feeling of psychological attachment and belonging that members exhibit toward their group (Tajfel and Turner, 1986; Huettermann et al., 2014). Scholars have claimed that group leadership is the main factor in shaping the group identification of members (van Knippenberg et al., 2004; Huettermann et al., 2014). Although no study has explored the relationship between ethical leadership and group identification, as drawn from SIT, this study argues that ethical leadership is expected to be positively related to group identification.

According to SIT, individuals would like to identify with a group that has distinct positive values (Ashforth and Mael, 1989); in seeking to establish positive differences between other groups and themselves, they try to enhance their self-esteem (Tajfel, 1982; Tajfel and Turner, 1986). Since ethical leaders instill and implement ethical standards and values in the group (Brown et al., 2005; Brown and Treviño, 2006), the groups display positive characteristics and values, such as justice, fairness, honesty, etc. These positive characteristics and values will foster the group identification of the members because they enhance their self-esteem. Group members are proud to identify with this kind of workgroup and they thus develop a high degree of identification. A leader’s clear ethical guidance fosters the perception of shared beliefs and norms (Zheng et al., 2015) and may also decrease the interpersonal conflicts among members (Mayer et al., 2012). According to SIT, the perception of shared beliefs and norms, or decreasing interpersonal conflicts, can be positively related to group formation and it can then promote group identification (Ashforth and Mael, 1989).

Drawing on SIT, this study expects that group identification is positively related to knowledge sharing, for the following two reasons: Firstly, when individuals identify with their group, they commit their efforts to supporting the group (Ashforth and Mael, 1989). In other words, as individuals with high collective identification, they will consider the collective interest as self-interest, and will intrinsically contribute to the collective good (van Knippenberg et al., 2004). Thus, when members within a group have a high degree of group identification, they will engage in knowledge sharing, since this kind of behavior is beneficial for the group. Secondly, SIT argues that social identification is helpful for forming intragroup cohesion, cooperation, and altruism (Ashforth and Mael, 1989). Therefore, members who identify with the group tend to engage in cooperative behavior, such as knowledge sharing. In addition, since the members evaluate the other group members with an altruistic and positive attitude, they will face less of a social dilemma about sharing their knowledge. Previous research has shown that developing an identification with a group is a useful way of dealing with social dilemmas (Zhang et al., 2014). Thus, they will be less likely to fear free riders and will be more willing to share their knowledge with others.

To sum up, the above explanations are consistent with SIT and the social identity model of leadership, which argue that leaders can motivate their followers to perform positive behavior by shaping the identification of their followers (Hogg, 2001; van Knippenberg and Hogg, 2003; Epitropaki et al., 2017). Therefore, it is reasonable to expect that ethical leaders will foster the group identification of their followers and, in turn, increase their knowledge-sharing behavior; therefore, this study posits the following:

Hypothesis 2: Group identification mediates the positive relationship between ethical leadership and knowledge sharing.

### Ethical Leadership, Relational Identification, and Organizational Identification

In this section, this study first explains how ethical leadership develops relational identification and, in turn, how it fosters organizational identification. The positive relationship between organizational identification and knowledge sharing is then illustrated. Finally, the series mediators, namely relational and organizational identification, are proposed to support the relationship between ethical leadership and knowledge sharing.

Although identification is considered as an important psychological mechanism and self-concept in organization research, most previous studies focus on individuals identifying with social groups (e.g., work groups or organizations) much more than work relationships. However, the work relationship plays an important role in the employees’ work environment; they rely heavily on good role relationships at work (e.g., subordinate-manager, coworker-coworker, and buyer-customer) to accomplish their daily tasks and to achieve a better work performance (Sluss and Ashforth, 2007). The role relationship of employees with their immediate supervisors is the most salient, because these supervisors in the workplace provide their employees with resources, or they punish them (Sluss and Ashforth, 2008). It is important to discuss the employee’s identification with the subordinate-manager role relationship; hence, the relationship identification that this study refers to is that an individual identifies with the subordinate-manager role relationship in a workgroup. Based on the definition of Sluss and Ashforth (2007), this study defines relational identification as the extent to which one defines oneself in terms of a given subordinate-manager relationship.

According to the relational identification theory (Sluss and Ashforth, 2007, 2008), as individuals enter a role relationship, the greater the perception of attractiveness or desirability of a relational identity, and the greater the development of relational identification. This study believes that some of the characteristics of ethical leadership benefit the establishment of a positive subordinate-manager role relationship. Since ethical leaders are considerate, honest and trustworthy (Brown and Mitchell, 2010), when they get along with their followers, their followers usually generate positive attitudes, such as satisfaction with their leaders and jobs (Ko et al., 2018). Therefore, this kind of role relationship is desirable for the followers. In addition, previous studies have shown that, under the guidance of ethical leaders, followers tend to perceive trust, task significance, and increased psychological capital and self-efficacy (Ko et al., 2018). In other words, followers can gain positive resources from the role relationship with an ethical leader. In summary, an ethical leader can make the subordinate-manager role relationship attractive and desirable to the followers, by associating it with the followers’ increased positive attitudes in the workplace and in their personal lives, and expanding their positive psychological resources. In a group, due to the salient and importance of this role relationship, members will tend to exhibit greater relational identification. Therefore, this study expects that ethical leadership is positively related to relational identification.

Following on the logic of the identification convergence perspective (Sluss and Ashforth, 2008), this study predicts that relational identification is positively related to organizational identification, which refers to the employees’ perception of unity with, and belonging to, their organization (Ashforth and Mael, 1989). The convergence of one’s different levels of self is explained by the notion of generalization, which occurs when an individual’s referent targets signify a resemblance (Sluss and Ashforth, 2008). In this study, it refers to two identifying referents simultaneously. Since role relationships and organizations, which are stimuli for relational identification and organizational identification, respectively, are structurally nested entities, they are logically considered as resembling each other. The convergence of relational identification and organizational identification occurs mainly *via* three mechanisms (Sluss and Ashforth, 2008; Sluss et al., 2012). Firstly, individuals with a high relational identification have a positive role relationship with their immediate supervisors (Sluss and Ashforth, 2007). Since role relationships with supervisors and organizations are easily linked together, the individuals will thus also have a positive effect on their organizations by forming organizational identifications. Secondly, individuals with a high relational identification tend to be easily influenced by their partners in the role relationship (Sluss and Ashforth, 2007). In this study, the relational partners of individuals are their supervisors, who are usually expected to speak positively about the organizations which, in turn, helps to increase the organizational identification. Thirdly, relational identification raises organizational identification through behavioral sense-making. Individuals identifying their role relationships with their supervisors will devote themselves to meeting the behavioral goals set by them. Since the goals of supervisors and the expectations of the organization are usually similar and overlap, when individuals achieve the behavioral goals of their supervisors, they also accomplish the behavioral goals of the organizations. Due to the need for self-consistency, individuals identify with the organizations through their behavior. According to the above three mechanisms, relational identification is expected to increase organizational identification, and this convergence of identifications (from relational to organizational identification) is empirically proven by two previous studies (Carmeli et al., 2011; Sluss et al., 2012). In summary, since ethical leadership is positively related to relational identification, and relational identification forms organizational identification, this study hypothesizes the following:

Hypothesis 3: Relational identification mediates a positive relationship between ethical leadership and organizational identification.

Furthermore, this study assumes that the organizational identification of members is positively related to their knowledge-sharing behavior. Organizational identification is related to the collective level of self and is one kind of social identification. According to SIT (Ashforth and Mael, 1989), when individuals identify with their organizations, they tend to generate in-group favoritism, and support the organizations. Researchers have shown that organizational identification is related to the extra-role behavior of employees (Riketta, 2005); therefore, when members identify with the organization, they are willing to conduct extra-role behavior, in order to benefit the organization. Basically, knowledge sharing is one kind of extra-role behavior (Wu and Lee, 2016), and therefore, organizational identification is supposed to increase knowledge sharing. In addition, when employees identify with the organization, they tend to put the collective interests (e.g., the organizational interest) before their own self-interest (van Knippenberg et al., 2004). Thus, they value the benefits of sharing knowledge with others more than hoarding knowledge for themselves. In other words, members with high organizational identification tend to perform extra-role behavior and consider the organizational interest, rather than self-interest as their first priority. Since knowledge-sharing behavior benefits the organization, this study assumes that members with high organizational identification would like to perform knowledge sharing.

Based on Hypothesis 3 and the abovementioned hypothetical relationship between organizational identification and knowledge sharing, this study proposes that there is a positive and indirect effect of ethical leadership on the members’ knowledge sharing *via* their relational and organizational identification. As a result, this study offers the following:

Hypothesis 4: Ethical leadership exhibits a positive, serial and indirect relationship with knowledge sharing *via* increased relational identification and, consequently, increased organizational identification.

## Materials and Methods

### Sample and Procedure

This study aims to explore the impact of ethical leaders on knowledge sharing. Thus, the moral issue of leaders is one of our concerns, as there have been some school scandals in Taiwan in recent years. For example, a school head stole school assets by making false claims. Such news shows that some leaders in Taiwanese schools have serious ethical problems. Basically, leaders in schools are supposed to be ethical leaders; however, in reality, this is not always the case. As a result, this study plans to use administrative groups in the schools as our research target.

The survey method was applied to this study by means of a questionnaire. Since this study explores the influence of ethical leadership on the knowledge sharing of followers, in the context of workgroups, the survey target in this study is the group members. The participants of this study are members of administrative groups of schools in Central and Northern Taiwan. Convenient sampling was used in this study. After the preliminary selection, the researchers contacted the schools *via* telephone to ask whether they were willing to participate in this study. Questionnaires were sent to the schools *via* delivery services, or in person, after confirming the number of administrative group members that could participate in the study. To ensure that the participants answered the questionnaire honestly, without worrying about identity exposure, all of the questionnaires had no unique reference numbers for identification.

A total of 600 administrative group members from 54 schools were invited to participate in this survey. A total of 510 participants completed the questionnaires successfully (an 85% response rate). Of the 510 participants, 63.3% were female, 70.5% were married, the average age was 39.56 years (*SD* = 8.03), the average tenure was 13.36 years (*SD* = 8.20), and 98.2% of the participants had an associate’s degree, or above.

### Measures

All of the measurements in this study used a seven-point scale. The response options were from 1 = “strongly disagree” to 7 = “strongly agree.” The back translation method was used to ensure that the meanings of items in the Chinese version were the same as the original items.

#### Knowledge Sharing

The scale developed by Lin (2007) was used to measure the members’ knowledge sharing, and it included four questions that were related to tacit knowledge sharing. The members were required to make assessments of their knowledge-sharing behavior. Samples of these items are as follows: “I share my job experience with my co-workers,” and “I share my expertise at the request of my co-workers.” The Cronbach’s α for this scale was 0.95.

#### Ethical Leadership

This study used the measurement items developed by Brown et al. (2005) for this scale. In total, there were 10 measurement items that were addressed by the group members to evaluate their perception of ethical leadership, for example, “Sets an example of how to do things the right way, in terms of ethics,” and “Defines success not just by the results, but also the way that they are obtained.” The Cronbach’s α for this scale was 0.95.

#### Organizational Identification

The organizational identification adopted the scale by Mael and Ashforth (1992). Sample items like “When someone criticizes (name of school), it feels like a personal insult,” and “I am very interested in what others think about (name of school)” were provided to the participants to evaluate their organizational identification. The Cronbach’s α for this scale was 0.89.

#### Relational Identification

The scale developed by Sluss et al. (2012) was applied to measure relational identification; it included four items that were offered to the group members, including: “My relationship with my immediate supervisor is an important part of who I am at work,” and “If someone criticized my relationship with my immediate supervisor, it would feel like a personal insult.” The Cronbach’s α for this scale was 0.86.

#### Group Identification

This study measured the extent to which group members identified with the workgroup, by using the same root items of organizational identification (Mael and Ashforth, 1992). This study adapted the identified referents from the organization to the workgroup. Sample items included: “When someone criticizes my workgroup, it feels like a personal insult,” and “I am very interested in what others think about my workgroup.” These were provided to the participants to evaluate their group identification. The Cronbach’s α for this scale was 0.94.

#### Control Variables

This study used the members’ demographic variables, such as gender, education, and working tenure, as the control variables. In addition, in order to reduce the negative effect of common method variance (CMV) on the results, as suggested by Podsakoff et al. (2003), the employees’ positive affect (Watson et al., 1988) was added to this study as one of the control variables of knowledge sharing. The Cronbach’s α for this scale was 0.96.

### Analytical Strategy

**FIGURE 2:**
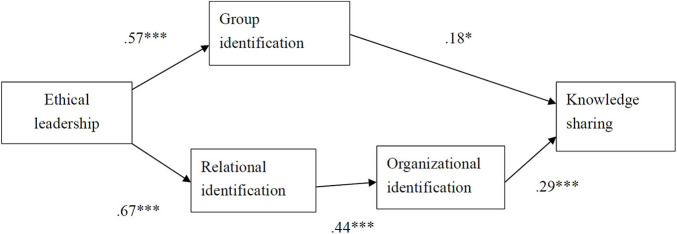
Results of path analysis. *p < 0.05, **p < 0.01, and ***p < 0.001.

This study used the SPSS package and Mplus 7 software to test the hypotheses. This study firstly employed a liner regression to test Hypothesis 1. Mplus 7 software was then used to verify the mediation effects. In order to test Hypotheses 2, 3, and 4, this study first depicted the path analysis model in Figure 2. This study then used the bootstrapping method (with 10,000 replications and 95% confidence intervals) in the Mplus program to test all of the indirect effect hypotheses. Regarding the control variables, because only positive affect had an impact on knowledge sharing (*b* = 0.18, *p* < 0.01), the influences of the control variables were omitted in Figure 2, in order to simplify the figure.

## Results

This study conducted a five-factor confirmatory factor analysis (CFA) model for the above five main measures (i.e., knowledge sharing, ethical leadership, organizational identification, relational identification, and group identification). Item parceling was used in the model for keeping a reasonable number of the degrees of freedom (Bandalos, 2002). The CFA results showed that this model achieved an acceptable fit, namely: GFI = 0.92, IFI = 0.97, CFI = 0.97, and RMSEA = 0.073. All of the measures had a composite reliability (CR) of above 0.82 and an average variance extracted (AVE) of above 0.70. The square roots of all the AVE scores were higher than any correlations of the possible focal pair measures. Therefore, both the convergent and discriminant validities were supported. In addition, as the main variables were filled out by team members, the CMV might influence the results. This study had conducted a Harman’s one-factor test to examine the CMV (Podsakoff et al., 2003), and the results showed that there were no serious problems regarding CMV in this study.

The descriptive statistics are presented in Table 1. To test Hypothesis 1, this study conducted a multiple regression model, as shown in Table 2. Hypothesis 1 predicts that ethical leadership has a positive effect on knowledge sharing. Model 1 of Table 2 shows that ethical leadership was positively and significantly related to knowledge sharing (*b* = 0.29, *p* < 0.001), thus Hypothesis 1 was supported.

**TABLE 1:** Means, standard deviations, and correlations.

Variables	Mean	S.D.	1	2	3	4	5	6	7	8
1. Gender^a^	0.37	0.48								
2. Education^b^	2.05	0.70	0.14**							
3. Working tenure	13.36	8.20	−0.06	−0.19***						
4. Positive affect	4.89	1.01	−0.01	−0.04	0.21***					
5. Ethical leadership	5.06	1.08	0.08	−0.03	0.06	0.52***				
6. Group identification	5.21	1.12	0.01	0.04	0.13**	0.57***	0.57***			
7. Relational identification	4.69	1.15	0.03	0.01	0.03	0.41***	0.67***	0.55***		
8. Organizational identification	5.19	1.06	−0.03	−0.05	0.20***	0.52***	0.54***	0.72***	0.44***	
9. Knowledge sharing	5.60	1.09	−0.04	−0.06	0.15**	0.48***	0.45***	0.53***	0.36***	0.55***

*p < 0.05, **p < 0.01, and ***p < 0.001.

*^a^1 = male, 0 = female.*

*^b^0 = senior high school, 1 = associate’s degree, 2 = bachelor’s degree, and 3 = master’s degree and above.*

**TABLE 2:** Result of regression analysis.

	Knowledge sharing
	**Model 1**
**Variables**	
Gender	−0.06
Education	−0.02
Working tenure	0.06
Positive affect	0.31***
Ethical leadership	0.29***
R^2^	0.29
F	41.83***

*p < 0.05, **p < 0.01, and ***p < 0.001.

Hypothesis 2 predicts that ethical leadership has a positive and indirect effect on knowledge sharing *via* group identification. As shown in Figure 2, ethical leadership was significantly and positively related to group identification (*b* = 0.57, *p* < 0.001), and group identification was significantly and positively related to knowledge sharing (*b* = 0.18, *p* < 0.05). The indirect effect of ethical leadership on knowledge sharing *via* group identification was 0.10 (*p* < 0.05), and the bootstrapping analyses showed that the 95% confidence interval did not contain zero (CI = [0.021, 0.178]). Thus, Hypothesis 2 was supported. Hypothesis 3 predicts that ethical leadership has a positive and indirect effect on organizational identification through relational identification. As shown in Figure 2, ethical leadership was significantly and positively related to relational identification (*b* = 0.67, *p* < 0.001), and relational identification was significantly and positively related to organizational identification (*b* = 0.44, *p* < 0.001). The indirect effect of ethical leadership on organizational identification *via* relational identification was 0.30 (*p* < 0.001), and the bootstrapping analyses showed that the 95% confidence interval did not contain zero (CI = [0.237, 0.354]). Thus, Hypothesis 3 was supported.

Hypothesis 4 predicts that ethical leadership has a positive influence on knowledge sharing through relational and organizational identification. As mentioned above, there was a positive and significant effect between ethical leadership and relational identification, and between relational and organizational identification. As shown in Figure 2, organizational identification was also significantly and positively related to knowledge sharing (*b* = 0.29, *p* < 0.001). The indirect effect of ethical leadership on knowledge sharing *via* relational identification and organizational identification was 0.09 (*p* < 0.001), and the bootstrapping analyses showed that the 95% confidence interval did not contain zero (CI = [0.047, 0.122]). Thus, Hypothesis 4 was supported.

## Discussion and Conclusion

This study is one of the first to explore the influence of ethical leadership on knowledge sharing through various identification mechanisms. Based on SIT, this study showed how ethical leadership fosters the identification of its followers and then enhances their knowledge sharing. First, the results of this study have shown that ethical leadership is positively related to employee knowledge sharing. This is consistent with previous studies (e.g., Lei et al., 2019; Su et al., 2021). Current studies have fully demonstrated the importance of ethical leadership in promoting employee knowledge sharing. Second, previous studies have found many mediators between ethical leadership and knowledge sharing, such as employees’ subjective well-being, social media interaction, positive reciprocity, moral efficacy, controlled motivation, moral identity, relational social capital etc. (Bavik et al., 2018; Abdullah et al., 2019; Bhatti et al., 2020; Su et al., 2021). This study further to prove that employees’ identifications are also important mediators. Specifically, this study demonstrated that ethical leadership has an indirect effect on knowledge sharing through increased group identification. Furthermore, ethical leadership exhibits a serial mediating effect on knowledge sharing *via* increased relational and organizational identification.

This study has some important theoretical contributions. Firstly, based on SIT, this study reveals how ethical leadership affects knowledge sharing by means of different kinds of identification. In the extant literature, researchers have proven that motivation, moral identity, trust, and culture are important mediators for the ethical leadership-knowledge sharing relationship (Bavik et al., 2018; Le and Lei, 2018; Lei et al., 2019). This study proves further that the employees’ perceptions of identification could translate into the influence of ethical leadership on knowledge sharing. In the workplace, employees rarely perform tasks or jobs alone, as they usually work within a workgroup. Therefore, it is important for employees to identify with the workgroup. This study demonstrates that ethical leadership has not only a direct effect on knowledge sharing, but it also has an indirect effect through increased group identification. This result is consistent with previous studies that group identification is an important psychological mechanism that connects leadership with the followers’ desired organizational behavior (Liu and Li, 2018). Moreover, this study examines the impact of ethical leadership on knowledge sharing *via* relational and organizational identification. This serial mediation effect not only echoes the argument that different types of identification might converge (Sluss and Ashforth, 2008; Sluss et al., 2012), but it also gives us a clearer understanding of the mechanism between ethical leadership and knowledge sharing. Although previous studies have found several mediators between ethical leadership and knowledge sharing, this study is the first one exploring the psychological mechanism on the ethical leadership-knowledge sharing relationship with a perspective of SIT. The results of this study depict a vivid picture of how different kinds of employee identification mediate the relationship between ethical leadership and knowledge sharing. It shows us that the identification of employees could be a promising psychological mechanism for the relationship between ethical leadership and knowledge sharing for future studies.

Secondly, introducing SIT into this study has expanded the scope of its application. More importantly, depending on the abundant research results of SIT in previous studies, it could offer many useful insights for future knowledge-sharing studies. For example, this study indicates that organizational identification is significantly related to knowledge sharing. Previous studies on SIT have already shown that organizational identification could be promoted from different perspectives, such as perceived organizational prestige (Carmeli et al., 2007) or support (Zagenczyk et al., 2011), etc. This study thus has a more theoretical ground for exploring how to increase the employees’ organizational identification, which, in turn, promotes knowledge sharing. Similarly, the results of this study also contribute to the social identity model of leadership (Hogg, 2001; Epitropaki et al., 2017). In this model, researchers argue that leaders could promote the followers’ positive behavior, depending on the shape of their identification. This study offers some evidence to support this model by showing that ethical leaders could motivate followers to perform knowledge sharing (positive behaviors) by shaping their group and organizational identification.

Our study has several important implications for managers. Firstly, the results indicate that ethical leadership has positive direct and indirect effects on knowledge sharing. It means that if managers could serve as ethical role models and ensure that their followers can work in a moral environment, it could effectively promote the followers’ knowledge sharing behavior. Also, ethical leadership can also play a role in reducing work-related stress (Zhou et al., 2015). Thus, it is important for companies to help their leaders to become good ethical leaders. For example, in order to improve the managers’ moral awareness, companies could offer more ethical training programs for their managers. Secondly, the relationship between group identification and knowledge sharing is significant. Managers could create a more positive atmosphere within the workgroup, or a higher group reputation, which could both help group members to have a higher level of group identification. Finally, our research has found that the relational identification of employees with their supervisors is an important mediator that translates ethical leadership into organizational identification, which, in turn, leads to knowledge sharing. In general, when a subordinate-manager relationship is more attractive or desirable, employees are more willing to identify with the role relationship. Thus, managers should keep in mind that it is important to build a positive and high-quality relationship with their subordinates, in order to increase their relational identification.

## Limitations

Some of the limitations of this study include the following: firstly, the hypotheses of this study imply that there is a causal relationship in nature. However, the survey has a cross-sectional design. Future studies could use a longitudinal design for collecting data, in order to have a rigorous sampling method. Secondly, the knowledge-sharing scale is rated by participants, but people sometimes might over-evaluate their positive behavior, such as knowledge sharing. Future studies might ask the participants’ coworkers or supervisors to fill out this scale. Thirdly, all of the measurement scales are self-reported. Although this study has added some control variables (e.g., positive affect) and conducted Harman’s one-factor test to ensure that there is no serious problem with the CMV, future studies could try to collect the data from multiple sources. Fourthly, according to SIT, there are different types of identification, but this study only includes three. Future studies could also include some other types of identification into their theoretical models, such as professional or personal identification. Furthermore, different types of identification may also interact or converge with one another, therefore future studies could further explore these rich and complex identity mechanisms between ethical leadership and knowledge sharing. Fifthly, since the sample in this study only includes the administrative group members of schools in Taiwan, it might cause an issue that would the results of this study could be inferred into other contexts, such as group members from different kinds of work groups. Future studies could further to verify the results of this study by test the hypotheses with different types of samples. Finally, since the main purpose of this study is to explore the identification mechanism between ethical leadership and knowledge sharing within the context of a workgroup, a participant in this study must be a member of a workgroup. In order to ensure that this study can collect enough samples, the method of convenience sampling was applied to this study. However, because convenience sampling is not a method that collects the data with random sampling, the results of this study might lack a good level of generalization. Therefore, even the results of this study give us an initial understanding of the identification mechanism on the ethical leadership-knowledge sharing relation, we still have to bear in mind that the results of this study need to be verified by more relevant studies in the future.

## Data Availability Statement

The raw data supporting the conclusions of this article will be made available by the authors, without undue reservation.

## Ethics Statement

The studies involving human participants were reviewed and approved by the Human Research Ethics Committee at National Cheng Kung University, Taiwan. Written informed consent for participation was not required for this study in accordance with the national legislation and the institutional requirements.

## Author Contributions

The author confirms being the sole contributor of this work and has approved it for publication.

## Conflict of Interest

The author declares that the research was conducted in the absence of any commercial or financial relationships that could be construed as a potential conflict of interest.

## Publisher’s Note

All claims expressed in this article are solely those of the authors and do not necessarily represent those of their affiliated organizations, or those of the publisher, the editors and the reviewers. Any product that may be evaluated in this article, or claim that may be made by its manufacturer, is not guaranteed or endorsed by the publisher.
